# Recombinant Viruses for Cancer Therapy

**DOI:** 10.3390/biomedicines6040094

**Published:** 2018-09-25

**Authors:** Daria S. Chulpanova, Valeriya V. Solovyeva, Kristina V. Kitaeva, Stephen P. Dunham, Svetlana F. Khaiboullina, Albert A. Rizvanov

**Affiliations:** 1 Institute of Fundamental Medicine and Biology, Kazan Federal University, Kazan 420008, Russia; daryachulpanova@gmail.com (D.S.C.); solovyovavv@gmail.com (V.V.S.); olleth@mail.ru (K.V.K.); sv.khaiboullina@gmail.com (S.F.K.); 2 School of Veterinary Medicine and Science, University of Nottingham, Sutton Bonington Campus, Leicestershire LE12 5RD, UK; Stephen.Dunham@nottingham.ac.uk; 3 Department of Microbiology and Immunology, University of Nevada, Reno, NV 89557, USA

**Keywords:** recombinant viruses, virus-based vaccines, oncolytic viruses, gene therapy, cell therapy, chimeric antigen receptor (CAR) T-cell therapy

## Abstract

Recombinant viruses are novel therapeutic agents that can be utilized for treatment of various diseases, including cancers. Recombinant viruses can be engineered to express foreign transgenes and have a broad tropism allowing gene expression in a wide range of host cells. They can be selected or designed for specific therapeutic goals; for example, recombinant viruses could be used to stimulate host immune response against tumor-specific antigens and therefore overcome the ability of the tumor to evade the host’s immune surveillance. Alternatively, recombinant viruses could express immunomodulatory genes which stimulate an anti-cancer immune response. Oncolytic viruses can replicate specifically in tumor cells and induce toxic effects leading to cell lysis and apoptosis. However, each of these approaches face certain difficulties that must be resolved to achieve maximum therapeutic efficacy. In this review we discuss actively developing approaches for cancer therapy based on recombinant viruses, problems that need to be overcome, and possible prospects for further development of recombinant virus based therapy.

## 1. Introduction

Cancers are the second most common cause of death worldwide. According to statistical data, the number of new cases increases every year despite significant improvements in existing therapy and the emergence of new innovative therapeutic approaches [1]. Surgery, chemotherapy, and irradiation are the mainstream therapeutic approaches used for cancer treatment; hormone and monoclonal antibody therapy are also actively used [2].

Recently, gene and cell therapies were introduced as having significant potential for cancer treatment. Gene therapy implies correction or modulation of the expression of a gene of interest in the host. It can be used to achieve both increased expression of immunomodulating or oncosuppressor genes as well as to inhibit oncogene expression leading to tumor regression [3]. Cell-mediated therapy uses autologous patient cells, such as T lymphocytes and dendritic cells (DC), to induce an immune response, or the use of tumor cells directly as tumor-specific antigen donors [4,5,6]. Due to natural tropism toward tumor sites, the therapeutic potential of mesenchymal stem cells (MSCs) as vectors for chemotherapeutic drug delivery is under extensive investigation [7]. Genetic modification of MSCs has also been investigated, enabling the delivery of genes with oncosuppressive, immunomodulory or pro-apoptotic properties [8]. However, cell-based therapy has a number of potential disadvantages, mediated by the properties of cells. For example, T-cells can induce cytokine release syndrome because of uncontrolled release of cytokines [9]. Use of MSCs, in turn, has a risk of unlimited cell growth, undesirable transformation and potential tumor formation [10].

Cell-free therapy is an alternative to cell-based therapy, which is based on the use of extracellular vesicles (exosomes and microvesicles) derived from native or genetically/chemically modified cells. The cell-free approach was developed in an attempt to circumvent the limitations of cell-based therapy. Extracellular vesicles (EVs) retain properties of the donor cells, however, more importantly, they can carry donor cell cargo or externally loaded content [10]. However, challenges remain in the use of EVs for cancer therapy. EVs can have variable effects on tumor progression dependent upon their cargo; heterogeneity of the isolated population and inconsistency of the EV cargo add an additional caveat to their study and therapeutic use [11].

Genetic modification of cells using recombinant viruses can promote high levels of transgene expression in a wide range of host cells [12]. With direct gene therapy, viral vectors, depending on the type of virus and genome modification employed, can serve as immunomodulating agents [13] or have direct oncosuppressive properties [14].

## 2. Recombinant Virus-Based Therapeutic Vaccines

**Figure 1:**
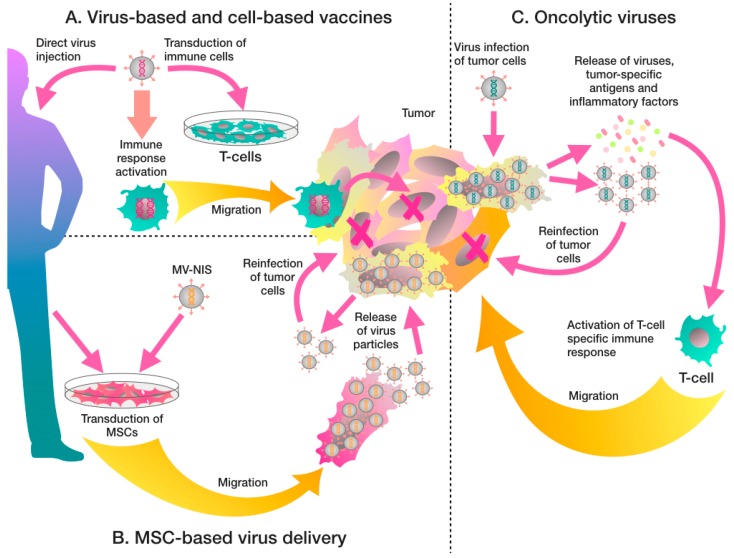
Recombinant virus based treatment of cancer. (**A**) Antitumor therapeutic vaccines, which are based on viruses encoding tumor-specific antigens, boost anti-cancer immune responses by enhanced presentation of the tumor antigens to immune cells. Another promising approach is the use of immune cell-based vaccines to stimulate antitumor immunity. In this case, T-cells are genetically engineered to express tumor-specific antigen receptors to improve recognition of cancer cells, for example CARs. (**B**) Therapeutic viruses, for example MV-NIS (the Edmonston strain of measles virus), can also be delivered to the tumor with mesenchymal stem cells (MSCs), which have a natural tropism toward tumor niches. (**C**) Oncolytic viruses preferentially infect tumor cells and induce tumor cell death. Additional genetic modification with immunomodulating genes such as granulocyte-macrophage colony-stimulating factor (*GM-CSF*), can enhance anti-tumor effect. Oncolytic viruses also cause local inflammation, which manifests as increased infiltration of immune cells into the tumor, local release of interferons (IFNs), chemokines, danger-associated molecular patterns (DAMPs), pathogen-associated molecular patterns (PAMPs) and mediate a tumor-specific immune response.

Recombinant viruses can be used to genetically modify cells, triggering the expression of tumor-specific antigens. These viruses can also be used to directly infect antigen-presenting cells to boost anti-cancer immune responses [15]. The enhanced presentation of the tumor antigens to immune cells leads to increased frequency and avidity of cytotoxic T-cells [16]. Additionally, transgenes expressed by the viral vector are more immunogenic than antigens administered with adjuvants [17]. Therefore, recombinant viruses present a valuable tool for development of antitumor therapeutic vaccines. The general mechanism of action of antitumor therapeutic vaccines is described in Figure 1A.

Tumor-specific antigens used for therapeutic vaccines should induce strong immune responses and be expressed only in tumor cells. Target specificity can result from antigens generated by somatic point mutations [18] or integrations of oncogenic virus genes [19] which are recognized by host T-cells. Cancer-testis antigens (CTAs), for example MAGE-A1 [20], are considered ideal targets for cancer vaccines due to their high immunogenicity and specific expression in germ and cancer cells [21,22]. Normal host antigens overexpressed by tumors may also be used as antigens for tumor immunotherapy. Carcinoembryonic antigen (CEA) is a glycoprotein that is often overexpressed in colorectal and other carcinomas, but also in some host cells. Its potential for therapeutic vaccination has been widely investigated, however additional strategies are needed to overcome immune tolerance, for example T-cell costimulation and blockage of CTLA-4 [23].

Several viruses have been used in attempts to develop optimal strategies for transgene delivery for a range of cancers. These include, retroviruses, lentiviruses (including HIV-1) [24], adenoviruses [25], adeno-associated viruses [26], poxviruses [27] and herpesviruses [28]. The choice of virus for a specific cancer is determined by virus tropism, the size of the transgene that needs to be packaged, desired longevity of transgene expression and potential safety concerns [29].

Poxviruses, which are able to induce both CD4^+^ and CD8^+^ mediated immune response against heterologous antigens, are the most widely used viruses for anti-tumor vaccine production [30]. Poxviruses have a large genome (130–230 kb) that allows for the introduction of large foreign DNA sequences. Several poxvirus vectors have been developed for gene therapy, including canary pox virus-based ALVAC, modified vaccinia virus Ankara (MVA), and NYVAC [31,32,33].

MVA has been used to generate a number of vaccines carrying specific antigens overexpressed in tumor cells including 5T4 [34], mucin 1 (MUC1) [35] and prostate-specific antigen (PSA) [36]. Typically, such vaccines have shown low toxicity in Phase I clinical trials and were highly immunogenic in some patients [37,38]. However, despite the stimulation of an antigen-specific immune response, vaccination did not lead to a significant clinical improvement in clinical trial participants. The lack of significant improvement in patients can be explained by the ability of the tumors to evade the immune system recognition [39]. Attempts to solve this problem have led to development of a number of viral vectors which carry not only tumor-specific antigen but also sequences of immunomodulating proteins or costimulatory molecules.

For example, TG4010 vaccine, which is an MVA encoding the MUC1 antigen as well as the human interleukin 2 (*IL-2*) gene, was shown to be safe in Phase I clinical trials. The Phase II clinical trial showed significant activation of MUC1 CD8^+^ specific immune response in more than half of the patients with PSA [40]. However, in patients diagnosed with metastatic clear cell renal carcinoma, treatment with MVA MUC1 vaccine combined with interferon α2a (IFN-α-2a) and IL-2 failed to establish long-term CD4^+^ and CD8^+^ immune responses [41] even though cytokine combination showed a statistically significant superiority in terms of response in renal carcinoma patients [42].

TRICOM poxviral vaccine encoding CEA and three costimulatory molecules B7.1, inter-cellular adhesion molecule-1 (ICAM-1), and lymphocyte function-associated antigen 3 (LFA-3 or CD58), was used to treat patients with CEA-expressing cancers in combination with IFN-α-2b. Administration of IFN-α-2b led to significant increase in overall survival (OS) compared to the vaccine alone [43]. Preclinical investigations using a mouse model with CT26-MUC1 tumors showed that combination of the TG4010 vaccine with immune checkpoint inhibitors anti-programmed cell death 1 (anti-PD-1)/programmed cell death ligand 1 (anti-PD-L1) resulted in delay of tumor onset as compared with TG4010 alone [44].

Multiple clinical trials have demonstrated the efficacy of combined therapy where various viral vaccines were used together with chemotherapy or radiotherapy. For example, TG4010 vaccination enhanced the effect of cisplatin and gemcitabine in a Phase IIB clinical trial in patients with non-small-cell lung cancer [45]. However, in another clinical study, the combination of modified vaccinia Ankara-5T4 with cyclophosphamide (CY) did not increase vaccine-mediated immune responses [37]. Injection of the TG4010 vaccine after a single 8 Gy radiation exposure increased the survival rate of RenCa-MUC1 cell-injected mice compared with vaccine, radiation, or radiation with MVA empty vector [46]. It appears that combined therapy has great potential which require further investigation.

In addition to the ability of tumors to evade immune surveillance, the natural ability of the host’s immune system to neutralize viral vaccines can limit the efficacy of viral vaccines, because booster doses do not increase antigen-specific immune responses [47]. Another disadvantage of antigen-specific vaccination using a single virus vector, is the likelihood that the immune response is mostly directed against highly immunogenic viral antigens, rather than tumor-specific antigens, which are often weak immunogens [48]. One approach to overcome this problem is to use two different recombinant viral vaccines that carry the same tumor-specific antigen to enhance tumor-specific immune responses. For example, the efficacy of multiple vaccinations (2–6 times) with MVA combined with simian adenovirus ChAdOx1 encoding mouse STEAP1 antigen was shown to be effective in the treatment of mice with prostate cancer. Vaccination with these viruses in combination with PD-1 blocking antibody significantly improved animal survival rate, and tumors were not observed in 80% of mice [49].

The idea of stimulating the host anti-tumor immune response by injecting viruses encoding tumor-specific antigens has been tested in large number of in vitro and in vivo studies, as well as clinical trials. However, patients, participating in these clinical trials, were already at the advanced stages of tumor development, when standard therapy was not effective. At these stages the immune system becomes significantly suppressed by tumor [50], leading to failure of induction of an adequate response to vaccination.

The large number of ongoing clinical trials (see Table 1) using viral vaccines combined with various therapeutic agents suggests that researchers remain hopeful of identifying effective drugs capable of significantly enhancing the immune response against tumors, despite the weakened immunity in cancer patients.

## 3. Virus-Based Engineering of Cell-Based Vaccines

The idea of using autologous ex vivo modified immune cells inspired new ideas in the field of antitumor vaccines. This approach has proven more successful and, currently, FDA has approved three immune cell-based vaccines to stimulate antitumor immunity. KYMRIAH (tisagenlecleucel) and YESCARTA (axicabtagene ciloleucel) are patient’s T-cells genetically modified using lentiviral or retroviral vectors, respectively, encoding anti-CD19 chimeric antigen receptor (CAR). YESCARTA was FDA approved in October 2017 for relapsed-refractory lymphoma (NCT02348216). KYMRIAH was approved for diffuse large B-cell lymphoma (DLBCL), TFL and high-grade B-cell lymphoma treatment after at least two lines of therapy on 1 May 2018 (NCT02445248). The third antitumor therapeutic vaccine PROVENGE (sipuleucel-T) was approved in 2010 for the treatment of prostate cancer. This vaccine is not subjected to direct genetic modification, instead, autologous antigen presenting cells (APCs) are cultivated in presence of recombinant human granulocyte-macrophage colony-stimulating factor (GM-CSF) and prostatic acid phosphatase (PAP), an antigen expressed in prostate cancer tissue [51]. All approved drugs for cancer therapy using recombinant viruses are summarized in Table 2.

The use of CAR T-cell approaches for immunotherapy is gaining momentum as evidenced by the number of active clinical trials. Few clinical trials have used virus-engineered DC-based vaccines to investigate the use of DCs, genetically modified to express various tumor-associated antigens, to induce antigen-specific immune responses. For example, adenovirus carrying the *P53* gene has been used to produce a DC-vaccine to promote anti-p53-specific immunity (NCT00049218, NCT00776295). Three active dose-escalation trials of MSCs are using viral infection to express *TRAIL* (NCT03298763), *IL-12* (NCT02530047) or *IFN-β* genes (NCT02079324). The number of clinical trials with viral vectors for cell-based vaccine production is shown in Table 1.

## 4. Oncolytic Viruses

The genetic modification of viral genomes, which was the basis for the development of viral vaccines, has become the framework for developing another approach for cancer therapy using oncolytic viruses. Oncolytic viruses are able to preferentially infect tumor cells directly, in contrast to viral vaccines, which are mainly used to modify the immune cells [17]. Most viruses can replicate more actively in cancer cells, because anti-viral immune responses, for example interferon production and apoptosis, are impaired in cancer cells [52]. To improve the specificity of viral oncolysis, viruses that are non-virulent in humans or genetically modified viruses can be used [14]. Genetic modification of viral genome also allows the introduction of genes to enhance immune response against tumor cells [53,54]. Oncolytic viruses typically enter their target cells following binding to specific host-cell surface receptors. For example, adenoviruses bind to coxsackie and adenovirus receptor, which is expressed on the surface of a large number of tumor cells [55]. Oncolytic viruses based on herpes simplex virus type 1 (HSV-1) successfully infect cells that carry on their surface herpesvirus entry mediator (HVEM), nectin-1 and 3-*O*-sulfate-modified heparan sulfate receptors, while HSV-2-based oncolytic viruses bind to HVEM, nectin-1 and nectin-2 receptors [56].

HSV-1-based viruses are the most commonly used viruses in oncolytic therapy. To date, there are three generations of these viruses, among them second and third generations have the most potent anti-cancer activity. The second-generation HSV-based virus G207 carries one mutation at both loci of *γ34.5* gene, encoding infected cell protein 34.5 (ICP34.5), which is necessary for HSV-1 infection of host cells. In addition, a disabling *lacZ* insertion in UL39 that encodes the large subunit of viral ribonucleotide reductase (RR), inactivates RR. This increases virus specificity for tumor cells (which have higher levels of endogenous RR, complementing viral RR deficiency) and thus enhances virus safety for non-malignant cells and tissues [57]. T-Vec (Imlygic, talimogene laherparepvec) is another second-generation virus, which was the first approved for clinical use. T-Vec comprises HSV-1 with deletions in *γ34.5* and *α47* genes (the latter encoding ICP47 protein), and human *GM-CSF* gene insertion, which increases the immunogenicity of the virus [53]. The third-generation HSV-based oncolytic virus G47Δ was obtained by introducing a third mutation into the HSV-1 G207 genome. In addition to the mutations in the *γ34.5* and *α47* genes, there is an insertion of *lacZ* gene into the *icp6* gene site leading to RR inactivation [58].

Vaccinia virus-based oncolytic virus JX-594 (Pexa-Vec, pexastimogene devacirepvec), in which the thymidine kinase (*tk*) gene is replaced with human *GM-CSF* and β-galactosidase (*β-gal*) genes, is also undergoing clinical trials [59]. To ensure selective replication in tumor cells and increase the safety, the *e1a* gene is commonly deleted in oncolytic adenoviruses [60,61]. These oncolytic viruses are discussed in more details in the review by Fukuhara et al. [14].

The ability of oncolytic viruses to induce tumor cell death was shown in vitro [62,63], in vivo [64] and in clinical trials [61]. Based on the large volume of accumulated data, a significant number of oncolytic viruses have reached the stage of clinical testing. In Phase I clinical trials, the safety of a number of oncolytic viruses, for example, HSV-1-based TBI-1401 (HF10) (NCT02428036) and HSV1716 (NCT01721018), and the ability of the JX-594 virus to replicate in tumor cells were demonstrated [65].

Prolonged patient survival is also shown in a number of clinical trials. For example, the one- and two-year median OS of patients with advanced head and neck cancer after treatment with oncolytic vaccinia virus GL-ONC1 was 86% and 76%, respectively, which is significantly higher than the known OS for conventional treatment (70% and 60%, respectively) (NCT01584284). An increase in median OS after administration of a large dose of JX-594 also indicates the effectiveness of therapy with oncolytic viruses (NCT00554372). A high success rate was achieved in clinical trials of T-Vec oncolytic virus. In a randomized Phase III trial (NCT00769704), patients with unresectable stage IIIB-IV melanoma were treated with T-Vec or subcutaneous human GM-CSF injection. The median OS in the human GM-CSF arm was 18.9 months, while in the T-Vec arm it was 23.3 months. The overall response rate in the arms was 5.7% and 26.4% of patients, respectively [66]. This data shows that T-Vac vaccine is effective in a proportion of patients with unresectable stage IIIB–IV melanoma.

In addition to the oncolytic effect, viruses cause local inflammation, which manifests with increased infiltration of immune cells into the tumor [60], local release of IFNs, chemokines, danger-associated molecular patterns (DAMPs) [67], pathogen-associated molecular patterns (PAMPs) and mediates a tumor-specific immune response [68]. The generation of tumor-specific immune responses by DAMPs and PAMPs has been comprehensively discussed elsewhere [69,70]. The lysis of cancer cells also leads to the release of tumor-specific antigens that can induce a broader antitumor CD8^+^ T-cell response. For example, administration of T-Vec to patients with metastatic melanoma increases the number of melanoma associated antigens recognized by T-cells (MART-1) specific T-cells as compared to controls [71]. A phase I study with ONCOS-102 oncolytic adenovirus showed induction of a systemic tumor antigen-specific T-cell response in 9 out of 12 participants, despite the heterogeneity of the patient population [60]. Interestingly, in a Phase II clinical trial, JX-594 oncolytic virus was able to infect tumor-associated endothelial cells, as well as cancer cells, ultimately obliterating tumor vasculature while leaving healthy vessels unaffected in patients with hepatocellular carcinoma (HCC) [72].

Oncolytic virus infection can trigger immune responses which suppress viral replication. The immune system produces neutralizing antibodies [61] and mobilizes innate immune cells [68] to restrict virus replication. If viruses are capable of inducing viremia, activation of an anti-viral immune response significantly reduces the therapeutic efficacy of oncolytic viruses. MV-NIS, the Edmonston strain of measles virus, which is genetically modified to express the human sodium-iodide symporter, specifically targets cells expressing high levels of CD46 on the surface. In a Phase I trial, MV-NIS was able to establish viremia, which led to production of neutralizing antibodies to MV-NIS in all patients [73]. To mask the virus from immune detection and limit viral immunogenicity, a cell-mediated viral delivery approach can be used. MSCs are promising cellular vectors because of their natural tropism to tumor sites [8]. Thus, human bone marrow-derived MSCs, infected with measles virus, migrated to orthotopically implanted HCC tumor cells and significantly suppressed tumor growth in both antibody-naïve and passively-immunized severe combined immunodeficiency (SCID) mice. In contrast, direct injection of virus had an anti-tumor effect only in antibody-naïve SCID mice [74]. The mechanism of MSC-based delivery of MV-NIS is shown in Figure 1B.

Most of the adverse effects associated with oncolytic vaccination are flu-like symptoms, such as fever/chills, rigors, nausea or vomiting [66,75]. Anemia [61], neutropenia, leukopenia and thrombocytopenia [73] are also reported, but only in some cases [61]. Unlike conventional drugs, many oncolytic viruses do not reach maximum tolerated dose (MTD), or dose limiting toxicity (DLT) [60,65,73]. Consequently, oncolytic virus-based treatment is commonly well tolerated.

Oncolytic virus-based therapy is also combined with chemotherapeutic drugs and human monoclonal antibodies. Potentially, combination of various treatment strategies could improve antitumor responses. For example, in a Phase Ib trial, T-Vec in combination with ipilimumab was used for melanoma treatment. Objective response rate in the combination arm was 39%, while in ipilimumab arm OR was 18% [76] (NCT01740297). The efficacy of T-Vec combination with Paclitaxel (NCT02779855) and JX-594 with the immune checkpoint inhibitor Durvalumab (NCT03206073) are also investigated in active clinical trials.

It is also worth mentioning oncolytic viruses which are not subjected to genetic modification. For example, wild type reoviruses predominantly replicate in tumor cells without affecting normal cells, targeting cells with an activated Ras pathway [77]. In a number of Phase II trials Reolysin (pelareorep), a reovirus based vaccine, was used in combination with various chemotherapeutic drugs (for example, gemcitabine, paxlitaxel, and cisplatin). Combined reovirus therapy has shown variable effects, having either a slight increase in median OS [78], or a delayed effect on the OS [79]. Reolysin showed a good therapeutic effect when combined with paclitaxel and carboplatin, in non-small cell lung cancer. This combination increased the objective response rate from 20% to 31% [80]. Another promising oncolytic virus is coxsackievirus A21 (CVA2, CAVATAK) which showed highly encouraging overall immune related progression free survival rate of 39% at six months in patients with stage IIIc and IV malignant melanoma (NCT01227551). The general mechanism of action of oncolytic viruses is shown in Figure 1C.

Oncolytic virus-based therapy is considered to be a breakthrough in cancer treatment. Indeed, direct lysis of tumor cells and induction of tumor-specific immunity may prolong patient survival rate. Suitable genetic modifications and combination with various chemotherapeutic drugs can be selected according to the type and stage of the cancer to achieve the most beneficial therapeutic effect. Many clinical trials (see Table 1) using various types of oncolytic viruses present the evidence that oncolytic viruses might be useful in combination therapy of malignancies.

## 5. Virus-Mediated Immunomodulation and Oncosuppression

In addition to cytotoxic effect and induction of tumor-specific immune response, recombinant viruses can directly modify tumor cells and the tumor microenvironment. For example, Rossowska et al. [81] used lentivirus (LV) encoding shRNA against TGF-β1 (shTGF-β1) to enhance the immunomodulatory effect of an antigen-primed dendritic cell (DC)-based vaccine. LV was injected into mice with murine colon carcinoma (MC38 cells) the day before the administration of the DC-based vaccine. However, the combination of LV-shTGF-β1 with DC-vaccine did not produce a significant effect. Cyclophosphamide used before the viral infection led to significant suppression of tumor growth compared to combined use of CY with DC-vaccine or LV-shTGF-β1 [81]. Co-delivery of TGF-β inhibitor SB-505124 and adenoviral vector carrying *IL-12* gene in B16 melanoma xenograft mice significantly delayed tumor growth and increased animal survival rate in vivo [82]. Lentiviral infection of breast cancer MCF-7 cells with USP39 shRNA induced G0/G1-phase arrest and apoptosis in vitro; the deubiquitinating enzyme USP39 plays important roles in mRNA processing [83].

Recombinant viruses can also be used to deliver tumor suppressor genes and apoptosis-inducing genes. For instance, lentivirus carrying human soluble tumor necrosis factor-related apoptosis-inducing ligand (*sTRAIL*) gene induced apoptosis in SGC-7901 cells in vitro by secreting bioactive sTRAIL protein [84]. In turn, recombinant adeno-associated virus (AAV)-encoding sTRAIL showed its safety in mice and in cynomolgus monkeys [85]. Interestingly, long-term expression of sTRAIL led to suppression of tumor growth in a mouse model with human SMMC-7721 liver cancer cells [86]. Combined therapy using adenovirus encoding phosphatase and tensin homolog (*PTEN*) gene, which is an oncosuppressor, and LY294002, the selective phosphatidylinositol 3-kinase (PI3K) inhibitor, led to significant induction of apoptosis in U251 glioma cells in vitro and in U251 subcutaneous glioblastoma xenograft model in vivo [87]. Adenoviral vector encoding *REIC/Dkk-3* gene suppressed the cell growth of human prostate adenocarcinoma LNCaP cells in vitro by inhibiting CD147 expression [88]. Thus, virus-mediated immunomodulation and oncosuppression can be used, either alone, or in combination with other therapeutic agents to increase its effectiveness.

## 6. Conclusions

Cancer therapy using recombinant viruses shows great promise and is currently being extensively investigated. The most studied approach includes using viral vectors as vaccines carrying tumor-specific antigens. These vaccines are aimed at stimulating the host’s immune system to suppress tumors and enhance T-cell-specific immune responses. Combination of these vaccines with immunomodulating agents is important to achieve the highest antitumor effect. It appears that the search for a successful combination of tumor antigen-bearing vaccines and other therapeutic agents will be the key to successful cancer treatment in a greater number of patients and cancer types.

Another breakthrough in virus-mediated cancer therapy is the use of oncolytic viruses, which have a direct cytotoxic effect on tumor cells, and additionally stimulate the immune response by releasing tumor-specific antigens and inducing local inflammation. Genetic modification of oncolytic viruses allows improvements in their specificity and immunogenicity to increase the effectiveness of therapy. As in the case of viral vaccines, combination with other therapeutic agents allows researchers to find the most effective approach for cancer treatment. This includes an FDA-approved oncolytic virus T-Vec which is successfully used to treat patients with melanoma.

In view of the fact that cancers are complex diseases that can arise as a result of various mutations, the approaches for the treatment of different cancers will vary. Most likely, the combination of various methods of treatment, including virus-mediated therapy, will lead to the most promising results in the fight against cancer.

## Figures and Tables

**Table 1:** A number or registered clinical trials of virus-based or virus-engineered therapeutic agents (according to clinicaltrials.gov).

	Clinical Trial Phase					
Therapeutic agent	I, I/II	II, II/III	III	IV	N/A *	Total
Oncolytic virus	73	31	7	0	4	115
Virus-based vaccine	39	38	0	0	0	77
Virus-engineered CAR T-cell	311	22	3	1	23	360
Other virus-engineered cell-based vaccines (DCs, MSCs)	11	4	0	0	0	15

* N/A, Not Applicable.

**Table 2:** FDA approved recombinant virus-based drugs.

Drug	IMLYGIC (Talimogene Laherparepvec, T-Vec), Oncolytic Virus	YESCARTA (Axicabtagene Ciloleucel), Genetically Modified Autologous T-cell	KYMRIAH (Tisagenlecleucel), Genetically Modified Autologous T-cell
Approval date	2015	2017	2018
Viral vector	HSV-1	Retrovirus	Lentivirus
Genetic modification	Deletions in γ34.5 and α47 genes and insertion of *GM-CSF* gene	Insertion of anti-CD19 CAR	Insertion of anti-CD19 CAR
Application	In patients with melanoma recurrent after initial surgery	Diffuse large B-cell lymphoma (DLBCL), TFL and high-grade B-cell lymphoma	B-cell precursor acute lymphoblastic leukemia (ALL), DLBCL, high grade B-cell lymphoma
Mechanism of action	Causes lysis of tumor, followed by release of tumor-derived antigens, which together with virally derived GM-CSF may promote an antitumor immune response	T-cell activation, proliferation, acquisition of effector functions and secretion of inflammatory cytokines and chemokines. This sequence of events leads to killing of CD19-expressing cells	Identify and eliminate CD19-expressing malignant and normal cells
Adverse reactions	Fatigue, chills, pyrexia, nausea, influenza-like illness, and injection site pain	Cytokine release syndrome, neurological toxicities, infections and febrile neutropenia, prolonged cytopenia, hypogammaglobulinemia	Cytokine release syndrome, neurological toxicities, infections and febrile neutropenia, prolonged cytopenia, hypogammaglobulinemia
Clinical studies	Randomized phase III trial (NCT00769704). Patients with stage IIIB–IV melanoma were injected with T-Vec or GM-CSF. OS in GM-CSF arm was 18.9 months, and T-Vec arm was 23.3 months; objective response in both arms was 5.7% and 26.4% of patients	In Phase II clinical trial (NCT02445248) efficacy was established based on complete remission (CR). Half of the patients achieved CR, while 21% achieved a partial response	ALL: In Phase II clinical trial (NCT02228096), efficacy of KYMRIAH was established based on complete remission (CR) within 3 months after infusion. Overall, 83% of patients achieved CR. DLBCL: In Phase II clinical trial (NCT02445248), efficacy was established based on complete response (CR) and partial response (PR). Overall 50% of patients achieved CR or PR
